# Prognostic value of the white blood cell-to-hemoglobin ratio (WBC/Hb) for inhospital mortality: Insights from a retrospective cohort study in Uganda

**DOI:** 10.1371/journal.pone.0340363

**Published:** 2026-01-20

**Authors:** Changhui Wu, Kuule Julius Kabbali, Kamugisha Richard, John Wanyama, Gemagaine Godfrey, Odong Christopher

**Affiliations:** 1 Department of Infectious Diseases and Hepatology, the Affiliated Hospital of Yunnan University, Kunming, Yunnan, China; 2 Department of Internal Medicine, Naguru Referral Hospital, Kampala, Uganda; 3 Department of Obstetric and gynecology, Naguru Referral Hospital, Kampala, Uganda; 4 Department of Pathology, Naguru Referral Hospital, Kampal, Uganda; King Fahd Military Medical Complex, SAUDI ARABIA

## Abstract

**Background:**

The white blood cell-to-hemoglobin ratio(WBC/Hb), composite marker derived from routine laboratory parameters, may offer unique prognostic value by integrating systemic inflammation and physiological reserve. This study investigates its association with inhospital mortality(IHM) in a resource-limited medical setting.

**Methods:**

We retrospectively enrolled patients admitted to medical ward of Naguru regional referral Hospital-Uganda, between January and June 2024. Data on demographics, clinical status, and lab results, including WBC-count and hemoglobin, were extracted on admission. The primary outcome was IHM. Patients were categorized into three WBC/Hb subgroups. Hazard ratios(HR) and Area Under the Curve(AUC) assessed its prognostic value, adjusting fully for age, sex, comorbidities, and admission diagnoses.

**Results:**

Overall, 226 patients were included(mean age 45.35 ± 18.85yrs, 54.4% female). The mean WBC/Hb ratio was 1.04 ± 1.22 × 10⁹ cells/L per g/dL, and IHM rate was 19.9%. Per-standard increase of WBC/Hb(2.22 × 10⁹ cells/L per g/dL) was associated with high-risk of IHM (HR 1.19, 95% CI 1.00–1.44; p = 0.012). The Results were similar when stratified into three groups (<0.55, 0.55–1.00, and ≥1.00 × 10⁹ cells/L per g/dL), compared with the reference group(<0.55), 0.55–1.00 group (HR 2.81, 95% CI 1.06–7.43; p = 0.037) and ≥1.00 group (HR 2.82, 95% CI 1.05–7.57; p = 0.040) had significantly high-risks of IHM. WBC/Hb demonstrated predictive value for IHM with AUC of 0.701 (95% CI 0.550–0.718).

**Conclusion:**

WBC/Hb, readily available and cost-effective marker, was associated with IHM. Incorporated into routine clinical assessments could improve risk stratification, especially in resource-limited settings. Prospective studies are needed to validate these findings and assess its broader utility.

## Introduction

Accurate and timely prognostication is fundamental for effective clinical decision-making, particularly in resource-limited settings where access to advanced diagnostics is constrained [1]. Among routine laboratory measurements, complete blood count(CBC) provides substantial clinical information [2]. Hemoglobin and white blood cell (WBC) counts [2,3] are independently recognized as biomarkers of disease severity and poor outcomes across a spectrum of illnesses, ranging from sepsis to hematologic disorders [3,4]. Despite this extensive evidence, the prognostic interaction between these two parameters specifically their ratio remains largely underexamined.

The ratio of WBC count to hemoglobin levels has been identified as a promising composite marker that reflects both inflammation and anemia [4,5], reflecting an individual’s capacity to respond to critical illness [4,6]. While WBC count is a well-established indicator of systemic inflammation, elevated in conditions such as infections, trauma, or malignancies [7], hemoglobin indicates the body’s ability to carry oxygen and its overall physiological health [4]. Taken together, WBC-to-hemoglobin(WBC/Hb) ratio may offer unique prognostic value [4], particularly in low-resource settings where sophisticated diagnostic modalities are unavailable [8,9]. However, evidence supporting its clinical utility remains sparse, and no study has systematically evaluated its prognostic relevance in low-resource health systems.

Uganda, like many low-and middle-income countries in the sub-Sahara Africa [8], bears an unnecessary burden of infectious diseases, trauma, and non-communicable illnesses [9], all of which contribute to high rates of in-hospital mortality(IHM). The adult HIV prevalence is about 5.8% among those aged 15–49, and tuberculosis incidence is roughly 198 cases per 100,000 people, resulting in about 94,000 new cases annually [10]. Malaria affects an estimated 12–16 million individuals each year, while non-communicable diseases account for around 36% of national deaths [11]. Inpatient case-fatality rates are 17.1% at Mulago National Referral Hospital and 15.4% for non-communicable diseases at a regional referral hospital [11]. In such environments, clinicians rely heavily on simple, low-cost tools for risk stratification [12]. Yet, despite its theoretical promise and accessibility, WBC/Hb ratio has not been rigorously studied as a prognostic marker in this context.

In this retrospective cohort study, we examine the association between the WBC/Hb ratio and IHM in a Uganda government-aided hospital care setting. By leveraging routine laboratory data, this study seeks to enlighten the potential of this ratio as a prognostic tool, providing insights that could inform clinical risk stratification and resource allocation in similar settings worldwide.

## Methods

### Ethics approval and consent to participate

The study utilized anonymized secondary data and did not involve direct interaction with participants. Consequently, the Institutional Review Board (IRB) of Naguru Regional Referral Hospital waived the requirement for informed consent, by national regulations. The study protocol was approved by the IRB of Naguru Regional Referral Hospital, in collaboration with the Ministry of Health of Uganda and the Uganda National Council for Science and Technology (UNCST) (Registration No: ADM/N/354/34/11/24).

### Study design and population

The study was a retrospective observational cohort analysis conducted at Naguru Regional Referral Hospital (NRH), focusing on adult patients admitted to the Internal Medicine department from January 1^st^ to June 30^th^, 2024. It included patients receiving care for various medical comorbidities, such as cardiovascular diseases, pneumonia, sepsis, malaria, anemia, HIV-related illnesses, and renal disease (Table 1). Data regarding clinical and demographic factors were extracted from the hospital’s information system for research purposes on September 2^nd^, 2024.

**Table 1:** Clinical and demographic characteristics.

Variables on admission	Overall (n=226)	WBC/ Hb Ratio/ (×10^9^ cells/L per g/dL)			P-*value*
		<0.55	0.55-1.00	≥1.00	
		76(33.6)	76(33.6)	74(32.7)	
Mortality, n(%)	45(19.9)	6(13.3)	19(42.3)	20(44.4)	**0.005**
WBC/ Hb Ratio/ 10^9^cells/L per g/dL	1.04 ± 1.22	0.40 ± 0.08	0.76 ± 0.14	1.99 ± 1.78	**<0.001**
Age, yrs	45.35 ± 18.85	44.96 ± 19.72	45.09 ± 17.86	46.03 ± 17.86	0.932
Gender, n(%)					0.979
Female	123(54.4)	41(33.3)	41(33.3)	41(33.3)	
Male	103(45.6)	35(34.0)	35(34.0)	33(32.0)	
Alcohol, n(%)					0.675
Ever drunk	98(43.4)	32(32.7)	36(36.7)	30(30.6)	
Never drunk	128(56.6)	44(34.4)	40(31.3)	44(34.4)	
Smoking, n(%)					0.142
Ever smoked	31(13.7)	6(19.4)	11(35.5)	14(45.2)	
SBP, mmHg	127.07 ± 33.69	128.41 ± 31.57	128.42 ± 31.04	124.31 ± 39	0.693
DBP, mmHg	75.94 ± 22.45	75.92 ± 22.50	77.87 ± 19.91	73.99 ± 24.86	0.573
Never Smoked	195(86.3)	70(35.9)	65(33.3)	60(30.8)	
**Laboratories values**					
Lymphocyte, 10^9^ cells/L	1.78 ± 2.24	1.34 ± 0.89	2.05 ± 3.26	1.94 ± 1.84	0.108
Monocyte, 10^9^ cells/L	0.69 ± 0.54	0.44 ± 0.24	0.699 ± 0.43	0.94 ± 0.74	**<0.001**
WBC, 10^9^cells/L	9.49 ± 6.30	4.97 ± 1.70	9.14 ± 3.41	14.50 ± 7.78	**<0.001**
Hb, g/dl	8.56 ± 3.88	12.38 ± 3.17	12.05 ± 3.47	8.56 ± 3.80	**<0.001**
Cr, mg/dl	132.31 ± 277.27	82.31 ± 78.68	121.02 ± 213.27	195.27 ± 420.84	**0.040**
GGT, IU/L	75.24 ± 123.76	79.34 ± 156.26	67.75 ± 65.27	78.70 ± 132.76	0.811
**Comorbidities**					
Septicemia, n(%)	40(17.7)	1(2.5)	7(17.5)	32(80.0)	**<0.001**
HTN, n(%)	67(29.6)	22(32.8)	23(34.3)	22(32.8)	0.984
Pneumonia, n(%)	28(12.4)	6(21.4)	10(35.7)	12(42.9)	0.293
T2DM, n(%)	52(23.0)	15(28.8)	18(34.6)	19(36.5)	0.678
Malaria, n(%)	39(17.2)	16(41.0)	14(35.9)	9(23.1)	0.336
HIV, n(%)	54(23.9)	15(27.8)	21(38.9)	18(33.3)	0.518
**Treatment medications**					
Cefixime, n(%)	3(1.3)	1(33.3)	2(66.7)	0(0.0)	0.371
Ceftriaxone, (%)	170(75.2)	47(27.6)	61(35.9)	62(36.5)	**0.004**
Levofloxacin, n(%)	21(9.3)	7(33.3)	8(38.1)	6(28.6)	0.878
Metronidazole, n(%)	112(49.6)	30(26.8)	37(33.0)	45(40.2)	**0.032**
Blood Transfusion, n(%)	48(21.2)	7(14.6)	7(14.6)	34(70.8)	**<0.001**

Data are presented as mean ± SD or as a percentage (unless otherwise indicated).

The characteristics of the study population (n = 226) are based on hospitalised patient at internal medical department stratified into quartiles(Q) of WBC/ Hb Ratio.

Abbreviations: SBP, systolic blood pressure; DBP, diastolic blood pressure; HG, Hemoglobin, WBC, White blood cells; HTN, hypertension; DM, diabetes mellitus; HIV, human immunodeficiency virus; HIV, Human acquired Virus; Hb, hemoglobin; Cr, creatinine**. Bold***,* statistically significant.

**Fig 1:**
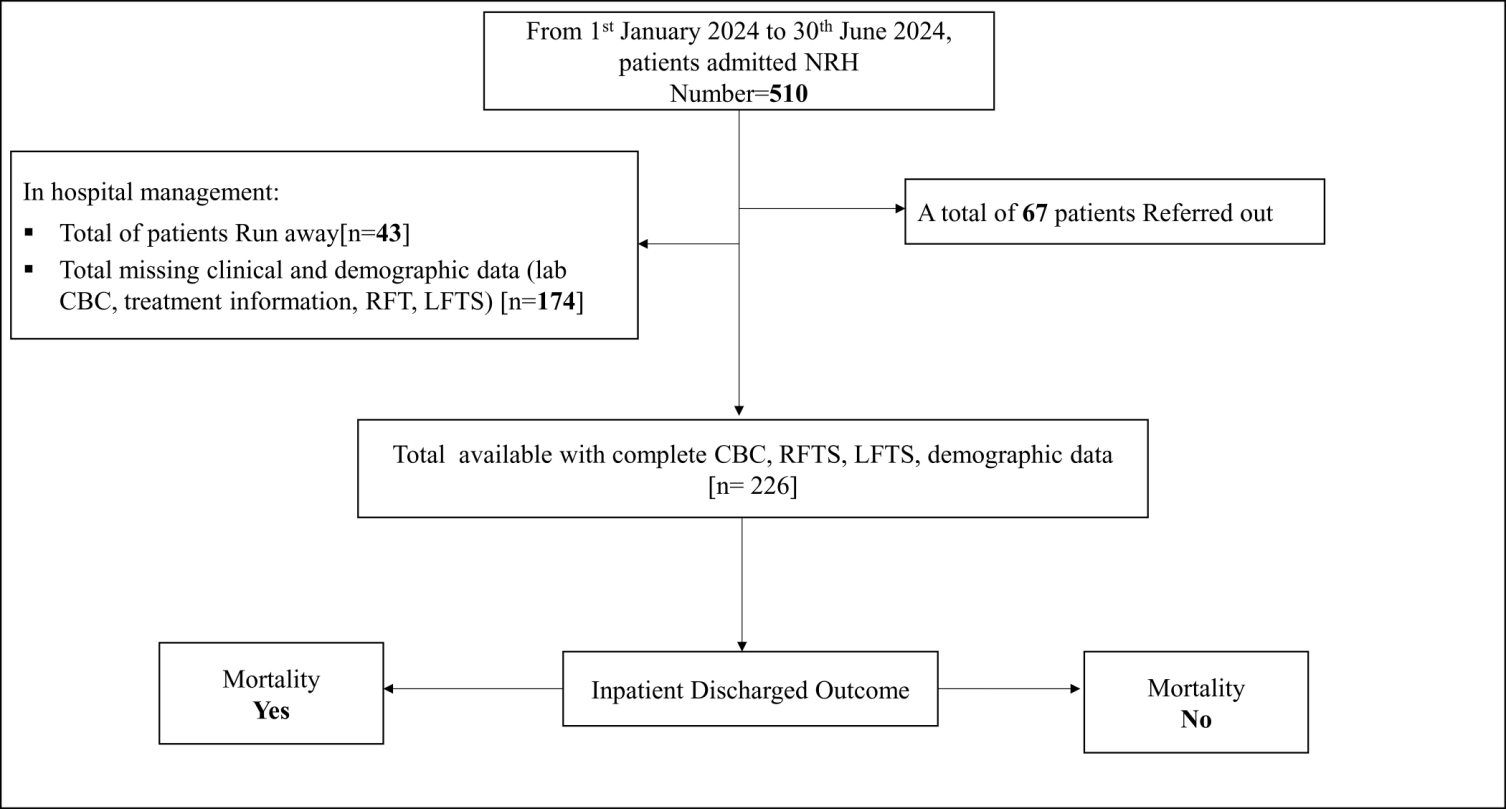
Flowchart Illustrating Patient Enrollment. Abbreviations: CBC, Complete Blood Count; RFTs, Renal Function Tests; LFTs, Liver Function Tests; NRH, Naguru Regional Referral Hospital.

A total of 510 patients received treatment in the medical ward at NRH during the first half of 2024. Of these, 67 patients referred-out for further management were excluded. Additionally, 217 patients with incomplete clinical, demographic, or discharge outcome data were excluded. The final study population included 226 patients with complete datasets, who were follow-up from admission to discharged, as shown in Fig 1.

### Study variables

The primary outcome variable was IHM [13], assessed as an adverse event. IHM was determined for patients hospitalized in the medical ward, based on official certificates or registration issued to confirm deaths occurring within the study period.

### Patient clinical assessment

Relevant characteristics potentially confounding the primary outcome were extracted from hospital records for the study period (January–June 2024). These characteristics included age, sex, smoking status, and alcohol consumption, with the latter being self-reported. Blood pressure was measured using a sphygmomanometer, and systolic (SBP) and diastolic (DBP) pressures were recorded as the average of three separate measurements [14]. Septicemia was identified through elevated lactate and/or C-reactive protein (CRP) levels, alongside clinical signs such as fever, tachycardia, hypotension, organ dysfunction, or positive blood cultures indicating bloodstream pathogens [15]. Pneumonia was diagnosed based on clinical features (e.g., fever, cough, and dyspnea), physical examination findings (e.g., crackles, abnormal breath sounds), chest imaging (e.g., X-ray demonstrating consolidation), and laboratory results (e.g., complete blood count, sputum cultures, and inflammatory markers) [16]. Malaria was confirmed by detecting *Plasmodium* parasites via microscopy, rapid diagnostic tests (RDTs), or blood smears (B/S) in symptomatic patients presenting with fever and/or chills [17]. HIV status was determined through self-reporting, antiretroviral (ARV) therapy records, or serological tests detecting HIV antibodies/antigens (e.g., ELISA or rapid tests) with confirmatory PCR testing [18]. The estimated glomerular filtration rate (eGFR) was calculated using the CKD-EPI formula, which incorporates serum creatinine levels, age, sex, and race to estimate kidney function [19]. Diabetes mellitus (DM) was defined by fasting blood glucose levels ≥126 mg/dL, random blood glucose levels ≥200 mg/dL, antidiabetic medication use, or a self-reported diagnosis by a physician [20]. Hypertension (HTN) was diagnosed based on systolic blood pressure ≥140 mmHg, diastolic blood pressure ≥90 mmHg, or the use of antihypertensive medication within two weeks before hospitalization [21]. Serum creatinine (Cr) levels were assessed using the kinetic Jaffe technique, an established method for clinical analysis [22].

### Statistical analysis

Statistical analyses were conducted using SPSS software version 25.0 (IBM, Armonk, NY, USA). Participant characteristics were stratified into three groups based on the WBC/Hb ratio: < 0.55, 0.55–1.00, and ≥1.00 × 10⁹ cells/L per g/dL. Hazard ratios and 95% confidence intervals (CIs) for IHM were calculated for these groups. Continuous variables were presented as mean ± standard deviation (SD), and categorical variables as counts and percentages. Hazard ratios (HRs) and 95% confidence intervals (CIs) for IHM were calculated for these groups. Sensitivity and specificity analyses were performed to evaluate the predictive utility of the WBC/Hb ratio for IHM. Multivariate regression models were employed, treating the WBC/Hb ratio as a continuous dependent variable. Three models were developed: Model 1 was unadjusted; Model 2 was adjusted for age and sex; Model 3 was fully adjusted, incorporating age, sex, smoking status, alcohol consumption, serum creatinine, ceftriaxone and metronidazole use, and history of blood transfusion. There were no missing data for variables included in the primary model. Proportional hazards assumptions for the Cox regression models were assessed using scaled Schoenfeld residuals and visual inspection of residual plots to ensure the validity of the hazard ratios. Linearity of continuous variables and influential observations were also checked to confirm model appropriateness. A subgroup analysis, displayed using a forest plot, was performed to identify high-risk subpopulations for IHM and to assess potential effect modification. Statistical significance was set at p < 0.05 with corresponding 95% CIs, and all tests were two-sided.

## Results

### Characteristics of the study population

A total of 226 participants admitted to the medical ward of NRH and receiving treatment were recruited for this study from January 1st to June 30th, 2024. The mean age of participants was 45.35 ± 18.85yrs, 54.4% were female, mean WBC/Hb ratio was 1.04 ± 1.22 × 10⁹ cells/L per g/dL. By the time of discharge, 19.9% of admitted patients had died. Participants were further categorized based on WBC/Hb ratio into three groups: < 0.55, 0.55–1.00, and ≥1.00 × 10⁹ cells/L per g/dL (Table 1).

**Fig 2:**
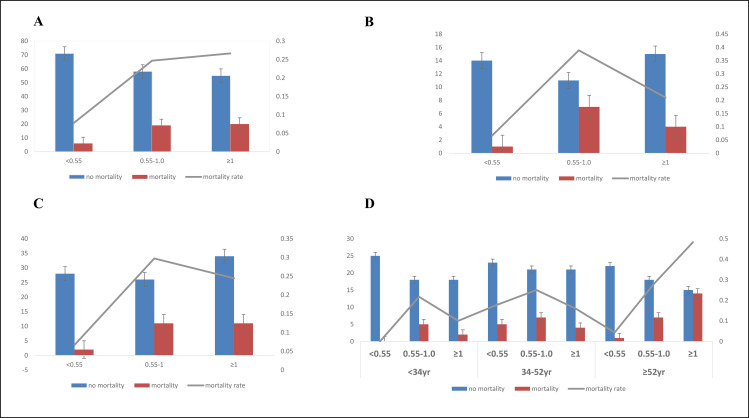
Incidence of IHM Across Stratified Groups (<0.55, 0.55–1.00, and ≥1.00 × 10^9^ cells/L per g/dL). (A) General Population, (B) Stratified by Diabetes Mellitus, (C) Stratified by Metronidazole Use, and (D) Stratified by Age.

The study population was categorized into three primary groups based on the WBC-to-Hb ratio: Group 1 included participants with WBC-to-Hb ratio of <0.55 × 10⁹ cells/L per g/dL, Group 2 consisted of those with a ratio between 0.55 and 1.00 × 10⁹ cells/L per g/dL, and Group 3 comprised participants with a ratio ≥1.00 × 10⁹ cells/L per g/dL. The distribution of participants across these groups was as follows: 33.6% in Group 1, 33.6% in Group 2, and 32.7% in Group 3(Table 1, Fig 2).

### WBC-to-Hb ratio and the risk of in-hospital mortality

The risk IHM increased with each standard elevation of 2.22 × 10⁹ cells/L per g/dL in the WBC/Hb ratio, with HR of 1.19 (95% CI: 1.00–1.44, *p* = 0.012). This association remained consistent across study subgroups. Using the WBC-to-Hb ratio of <0.55 × 10⁹ cells/L per g/dL as the reference group, the 0.55–1.00 × 10⁹ cells/L per g/dL ratio subgroup demonstrated an HR of 2.81 (95% CI: 1.06–7.43, *p* = 0.037), and the ≥ 1.00 × 10⁹ cells/L per g/dL ratio subgroup exhibited an HR of 2.82 (95% CI: 1.05–7.57, *p* = 0.040) in the fully adjusted model(Table 2, Fig 2).

**Table 2:** White Blood Cell-to-Hemoglobin Ratio and less than 30 days in In-Hospital Mortality.

WBC/ Hb Ratio/ (*10^9^cells/L per g/dL)	Model ^(*)^		Model 2^(**)^		Model 3^(***)^	
	HR(95%Cl)	P value	HR (95%Cl)	P value	HR (95%Cl)	P value
<0.55	Reference = 1		Reference = 1		Reference = 1	
0.55-1.0	3.20(1.256-8.163)	0.015	3.00(1.179-7.615)	0.021	2.81(1.06-7.433)	0.037
≥1.00	3.33(1.327-8.352)	0.10	3.09(1.229-7.775)	0.016	2.82(1.05-7.570)	0.040
Per-SD 2.22*10^9^ cells/L per g/dL elevation of WBC/ Hb Ratio	1.24(1.042-1.486)	0.016	1.17(1.987-2.394)		1.19(1.00-1.440)	0.012
Per-SD 6.30*10^9^ cells/L elevation of WBC	1.51(1.179-1.940)	<0.001	1.54(1.183-2.002)	<0.001	1.65(1.245-2.189)	<0.001
Per-SD 3.88 g/dL elevation of HG	0.69(0.507-9.38)	0.018	0.65(0.478-0.882)	0.006	0.55(0.359-0.852)	0.007

HR indicates, Hazard ratio.

(*^)^ Unadjusted.

(**^)^ Adjusted for age, and sex.

(***^)^ Adjusted for age, gender, smoking, alcohol, serum creatinine, Ceftriaxone, metronidazole, and Blood transfusion.

Proportional hazards assumptions were evaluated using scaled Schoenfeld residuals, the Grambsch and Therneau test, and visual inspection of Schoenfeld residual plots. No violations were detected, as indicated by the global test (p = X) and all covariate p-values greater than 0.05.

### WBC cell count, Hb level, and the risk of in-hospital mortality

**Fig 3:**
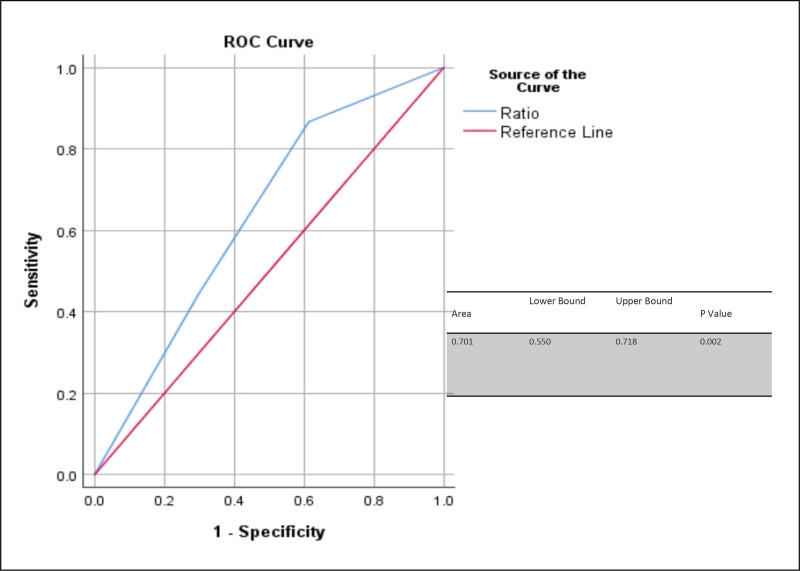
ROC Demonstrating the Predictive Value of the WBC/Hb Ratio for IHM. Abbreviation: ROC, Receiver Operating Characteristic; WBC/Hb Ratio, White blood cell to hemoglobin Ratio; IHM, In-Hospital Mortality.

Per-SD increase of 6.30 × 10⁹ cells/L in WBC count was associated with 1.65-fold increased risk of IHM (HR: 1.65, 95% CI: 1.245–2.189, *p* < 0.001). Conversely, per-SD increase of 3.88 g/dL in Hb level was associated with a 0.55-fold reduction in the risk of 30-day IHM, indicating a protective effect (HR: 0.55, 95% CI: 0.359–0.852, *p* < 0.001).Furthermore, WBC/Hb demonstrated predictive value for IHM with an AUC of 0.701 (95% CI 0.550–0.718) (Table 2, Fig 3).

### Sensitivity

**Fig 4:**
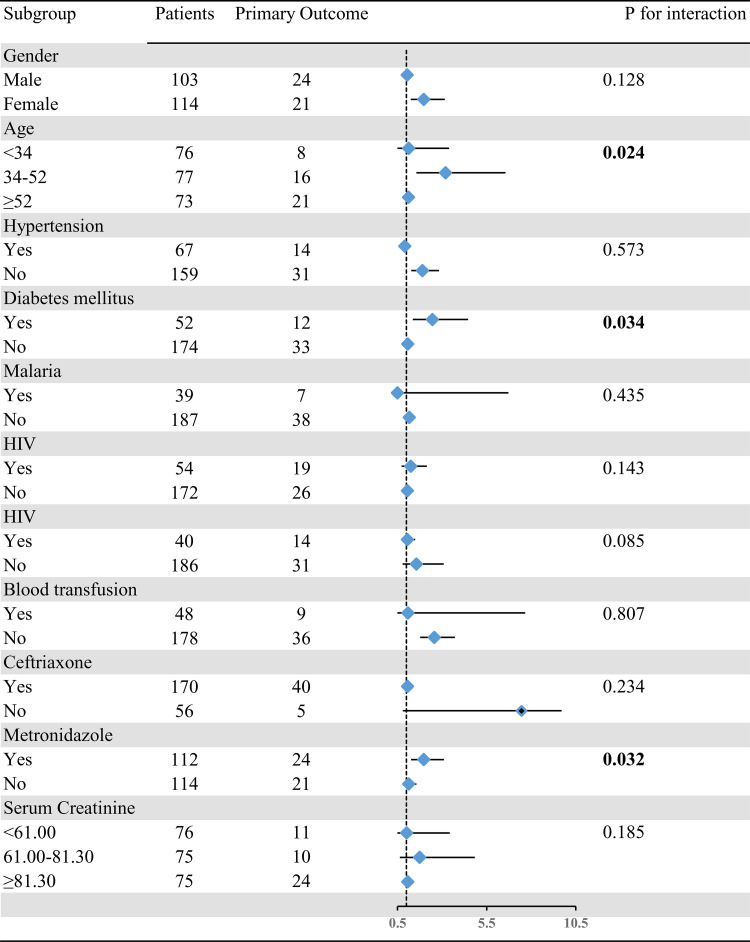
Forest Plot Depicting Subgroup Analysis of In-Hospital Mortality among High-Risk Groups.

A forest plot summarized prespecified risk factors for potential interactions related to in-hospital mortality and served as a sensitivity analysis to assess effect modification. All analyses were adjusted for age, gender, smoking status, alcohol consumption, serum creatinine levels, ceftriaxone use, metronidazole use, and history of blood transfusion, with each variable excluded in its respective subgroup comparison. Variables were categorized as follows: gender (male or female), age (<34, 34–52, ≥ 52 years), diabetes (yes or no), hypertension (yes or no), malaria (yes or no), HIV (yes or no), sepsis (yes or no), blood transfusion (yes or no), ceftriaxone use (yes or no), metronidazole use (yes or no), and serum creatinine (elevated or normal). Significant interactions were observed for age (P = 0.0324), diabetes mellitus (P = 0.034), and metronidazole use (P = 0.032) (Fig 2, Fig 4). These subgroup analyses confirm the consistency of the primary findings across key patient groups and demonstrate how specific factors modify the observed associations, thereby supporting the robustness and interpretability of the results.

## Discussion

This study explored the association between WBC/Hb ratio and the risk of in-hospital mortality among 227 patients admitted to the medical ward of NRH in Uganda. A key finding was the significant relationship between elevated WBC/Hb ratios and an increased mortality risk. Especially, for every standard increase of 2.22 × 10⁹ cells/L per g/dL in the WBC/Hb ratio, the risk of mortality rose by 1.19 times, even after adjusting for potential confounders such as age, gender, comorbidities, and treatment variables. Further analysis revealed that patients in the WBC/Hb ratio categories of 0.55–1.00 and ≥1.00 × 10⁹ cells/L per g/dL were over 2.8 times more likely to die compared to those in the reference group (<0.55 × 10⁹ cells/L per g/dL). This highlights a dose-dependent relationship between WBC/Hb ratio levels and mortality risk. Furthermore, elevated WBC counts independently predicted a higher risk of mortality (HR: 1.65), whereas higher hemoglobin levels were protective, significantly reducing mortality risk (HR: 0.55).

These findings emphasize the WBC/Hb ratio’s potential as a simple yet powerful prognostic marker in clinical practice, particularly in resource-constrained settings common in sub-Saharan Africa. The ratio provides a consolidated measure reflecting both inflammation (via WBC count) and oxygen-carrying capacity (via hemoglobin levels), making it especially valuable in populations where access to advanced diagnostic tools may be limited.

Our study is among the first to emphasize the clinical relevance of the WBC/Hb ratio in predicting short-term in-hospital mortality among the Uganda population. While previous studies have individually explored WBC count and hemoglobin levels as markers of inflammation and hypoxia, respectively [4,23], the combination of these parameters into a single ratio offers a novel approach [5,6,24]. This ratio provides an integrative measure that reflects both systemic inflammation and oxygen-carrying capacity, potentially capturing a broader picture of a patient’s physiological state [4]. Additionally, the stratification of patients into three WBC/Hb ratio groups allows for a nuanced understanding of risk levels, which could guide more tailored interventions [24].

Our findings are compatible with previous research demonstrating that leukocytosis and anemia independently predict poor outcomes across diverse patient populations [4,6]. Elevated WBC counts are well-established markers of systemic inflammation and are frequently associated with conditions such as sepsis, malignancies, and cardiovascular diseases each of which significantly contributes to IHM [4,6,7]. Similarly, anemia has been widely correlated to adverse clinical outcomes, primarily due to its effects on inadequate oxygen delivery to tissues and the potential for hemodynamic instability [25,26]. The observed protective association of higher hemoglobin levels (HR: 0.55) corroborates findings from cardiovascular and critical care studies, where hemoglobin optimization has been shown to improve survival outcomes [7]. Notably, our study contributes to the existing literature by demonstrating that the combined WBC-to-hemoglobin ratio may provide superior risk stratification compared to either parameter individually [7].

In our study, WBC/Hb ratio demonstrated an AUC of 0.701, which reflects moderate discriminative ability. This finding is significant as it extends the utility of the WBC/Hb ratio beyond earlier research [27], which primarily focused on specific groups like those with pulmonary hypertension or heart failure, and reported AUC values between 0.646 and 0.751 for the WBC/Hb ratio in predicting 30-day mortality [28]. In addition to the WBC/Hb ratio, the simple hematologic markers, such as the neutrophil-to-lymphocyte ratio (NLR), have also shown modest predictive capabilities with AUCs typically falling between 0.67 and 0.70 across various clinical contexts [29]. Our study’s diverse population, encompassing both acute and chronic medical conditions, underscores the WBC/Hb ratio’s usefulness as a practical indicator of illness severity. This marker relies on standard complete blood count parameters, making it particularly beneficial in resource-limited settings or situations demanding quick bedside assessments. Although it is not meant to replace comprehensive clinical scoring systems [30]. The WBC/Hb ratio may effectively complement these systems to aid in early risk stratification among varied patient populations.

Our study’s findings demonstrate important clinical relevance. The WBC/Hb ratio, a simple parameter derived from routine laboratory tests, may serve as an accessible prognostic indicator [7]. In settings with limited resources, where advanced diagnostic tools are unavailable, this ratio can facilitate early risk stratification by enabling clinicians to identify patients at increased risk of adverse outcomes [9]. This approach supports prioritization for enhanced monitoring, more frequent clinical assessments, or earlier initiation of supportive therapies [24]. Patients with elevated WBC/Hb ratios may require higher levels of care, expedited diagnostics, or timely anti-inflammatory treatment and correction of anemia using transfusions or erythropoiesis-stimulating agents. This ratio also highlights the importance of addressing both inflammation and anemia in hospitalized patients. Incorporating this parameter into routine clinical workflows may provide a cost-effective approach to improving patient management and survival outcomes [28]. As a simple and accessible biomarker, an elevated WBC/Hb ratio can help identify patients at higher risk of in-hospital mortality and support closer monitoring and timely, targeted interventions.

When used alongside tools like SOFA or NEWS scores [27], WBC/Hb ratio can provide additional information that enhances early risk stratification, especially in diverse medical population. Instead of acting as a substitute for validated scores like SOFA or NEWS, its strength lies in offering a quick and easily obtainable marker from routine laboratory tests. This is particularly useful in low-resource settings or situations where comprehensive scoring systems cannot be fully applied. By providing extra insight into a patient’s inflammatory and hematologic status, the WBC/Hb ratio has the potential to support existing prognostic frameworks rather than replace them.

## Study limitations

While our study provides valuable insights, several limitations warrant consideration. First, the single-center design may limit the generalizability of our findings to other populations with different demographic or clinical characteristics. Multi-center studies are necessary to validate our results in diverse settings. Second, the observational nature of our study precludes causal inferences. Although we adjusted for multiple confounders, residual confounding may still influence our findings. Further, we did not assess dynamic changes in the WBC/Hb ratio during hospitalization, which could offer further prognostic information. Third, the study period of six months might not fully capture seasonal variations in disease prevalence and outcomes, such as malaria or respiratory infections, which could affect WBC and hemoglobin levels. Extending the study duration could provide a more comprehensive understanding of the WBC/Hb ratio’s prognostic value. Furthermore, the interpretation of our findings, particularly the interactions involving age, diabetes, and metronidazole use, should be cautious due to the limited sample size of 226 patients with only 45 events (deaths). This lowers the statistical power and increases the chances of both type I and type II errors. Therefore, these results should be viewed as exploratory and validated in larger cohorts. To enhance the robustness of analyses in small datasets, advanced statistical methods like Firth’s regression or bootstrapping could be beneficial. Lastly, our study did not include detailed analyses of specific causes of death, which could provide insights into whether certain conditions, such as sepsis or hypovolemia, disproportionately influence the prognostic utility of the WBC/Hb ratio.

## Conclusion

Our study highlights WBC/Hb ratio as a novel and clinically significant marker for predicting in-hospital mortality. By integrating measures of systemic inflammation and oxygen-carrying capacity, this parameter offers a comprehensive risk stratification tool that is both accessible and practical. Our findings support the incorporation of the WBC/Hb ratio into routine clinical assessments, particularly in resource-constrained settings. However, further research, including prospective multi-center studies and interventional trials, is necessary to validate and refine its clinical utility.
